# Transposable elements impact the population divergence of rice blast fungus *Magnaporthe oryzae*

**DOI:** 10.1128/mbio.00086-24

**Published:** 2024-03-27

**Authors:** Lianyu Lin, Ting Sun, Jiayuan Guo, Lili Lin, Meilian Chen, Zhe Wang, Jiandong Bao, Justice Norvienyeku, Dongmei Zhang, Yijuan Han, Guodong Lu, Christopher Rensing, Huakun Zheng, Zhenhui Zhong, Zonghua Wang

**Affiliations:** 1 State Key Laboratory of Ecological Pest Control for Fujian and Taiwan Crops, College of Life Science, Fujian Agriculture and Forestry University, Fuzhou, China; 2 Fuzhou Institute of Oceanography, Minjiang University, Fuzhou, China; 3 State Key Laboratory for Managing Biotic and Chemical Treats to the Quality and Safety of Agro-Products, Institute of Plant Protection and Microbiology, Zhejiang Academy of Agricultural Sciences, Hangzhou, China; 4 Key Laboratory of Green Prevention and Control of Tropical Plant Diseases and Pests, Ministry of Education, College of Plant Protection, Hainan University, Haikou, China; 5 Institute of Environmental Microbiology, College of Resource and Environment, Fujian Agriculture and Forestry University, Fuzhou, China; 6 Ministry of Education Key Laboratory for Bio-Resource and Eco-Environment, College of Life Sciences, Sichuan University, Chengdu, China; Cornell University, Ithaca, New York, USA

**Keywords:** transposable element, population divergence, rice subspecies adaptation, rice blast disease

## Abstract

Dynamic transposition of transposable elements (TEs) in fungal pathogens has significant impact on genome stability, gene expression, and virulence to the host. In *Magnaporthe oryzae*, genome plasticity resulting from TE insertion is a major driving force leading to the rapid evolution and diversification of this fungus. Despite their importance in *M. oryzae* population evolution and divergence, our understanding of TEs in this context remains limited. Here, we conducted a genome-wide analysis of TE transposition dynamics in the 11 most abundant TE families in *M. oryzae* populations. Our results show that these TEs have specifically expanded in recently isolated *M. oryzae* rice populations, with the presence/absence polymorphism of TE insertions highly concordant with population divergence on Geng/*Japonica* and Xian/*Indica* rice cultivars. Notably, the genes targeted by clade-specific TEs showed clade-specific expression patterns and are involved in the pathogenic process, suggesting a transcriptional regulation of TEs on targeted genes. Our study provides a comprehensive analysis of TEs in *M. oryzae* populations and demonstrates a crucial role of recent TE bursts in adaptive evolution and diversification of the *M. oryzae* rice-infecting lineage.

**IMPORTANCE:**

*Magnaporthe oryzae* is the causal agent of the destructive blast disease, which caused massive loss of yield annually worldwide. The fungus diverged into distinct clades during adaptation toward the two rice subspecies, Xian/*Indica* and Geng/*Japonica*. Although the role of TEs in the adaptive evolution was well established, mechanisms underlying how TEs promote the population divergence of *M. oryzae* remain largely unknown. In this study, we reported that TEs shape the population divergence of *M. oryzae* by differentially regulating gene expression between Xian/*Indica*-infecting and Geng/*Japonica*-infecting populations. Our results revealed a TE insertion-mediated gene expression adaption that led to the divergence of *M. oryzae* population infecting different rice subspecies.

## INTRODUCTION

Rice blast disease, caused by the ascomycete filamentous fungus *Pyricularia oryzae* (syn: *Magnaporthe oryzae*), poses a significant threat to rice production worldwide, resulting in annual yield losses of 10%–30% (1, 2). The deployment of resistant rice varieties is the most cost-effective and environmentally friendly strategy for controlling rice blast disease. However, the effectiveness of such resistance can rapidly be diminished due to rapid mutation accumulation in avirulence genes (3, 4). Therefore, it is crucial to uncover the mechanisms by which *M. oryzae* rapidly evolves and evades the rice immune system.

As per the two-speed genome model, filamentous fungi genomes had been shown to display a bipartite architecture comprising of a gene-rich compartment, which evolves slowly containing core genes encoding essential functions and metabolisms, and a repeat-rich compartment, which evolves rapidly containing important virulence effectors involved in pathogenicity (5–10). To avoid recognition by the plant immune system, some pathogen effectors such as avirulence genes were shown to be influenced or silenced by transposon elements (9, 11). Transposable elements (TEs) make up over 10% of the *M. oryzae* genome (12, 13). Recent evidence has revealed that TEs can be inserted in or around important effectors and alter the virulence spectrum of *M. oryzae*. For example, the insertion of POT3 into the promoter region of Avirulence gene AVR-Pita in *M. oryzae* led to the acquisition of virulence towards the resistant rice cultivar Yashiro-mochi (14). TEs are also able to affect gene expression networks, and TE-dependent transcriptional regulation of some essential effectors can facilitate the pathogen’s transition in its life cycle. For instance, in *Phytophthora sojae*, the avirulence genes *PsAvr1a*, *1b*, and *3a/5* were found to be transcriptionally inactive due to TE insertions in their promoter or 3′ UTR (untranslated region) (15).

These studies have demonstrated the crucial role of TE-mediated genomic variations in pathogen adaptation. However, previous investigations have mainly focused on the impact of TEs on specific effectors or secreted proteins (SPs) (13, 16–20). To gain a better understanding on the functions of TEs in the complex *M. oryzae*-rice pathosystem, we conducted a comprehensive analysis of TE insertion polymorphisms in 275 *M*. *oryzae* isolates (176 rice isolates and 99 non-rice isolates; Table S1), and systematically investigated the roles of TEs in the regulation of gene expression and population divergence in the *M. oryzae* rice population.

## RESULTS

### Recent large-scale TE bursts in the *M. oryzae* genome

To assess the activity of TEs on the *M. oryzae* genome, we estimated the insertion time of long terminal repeat (LTR)-retrotransposons (LTR-RTs) by measuring the genetic distance between their 5′ and 3′ LTRs, which were identical at the time of TE insertion and gradually accumulated mutations over time. Using a *de novo* method that is based on the structure of LTR-RTs, we identified 1,129 intact LTR-RTs in seven near chromosomal-level *M. oryzae* rice isolates, including 70–15, Guy11, FJ81278, FJ98099, FJ72ZC7-77, AV1-1-1, and Sar-2–20-1 (12, 13, 21). Surprisingly, 91.7% (1,036/1,129) of the LTR-RTs showed extremely low levels of divergence between their LTR pairs (over 99% identity), and 69.4% (784/1,129) of them possessed identical LTR pairs, indicating that these LTR-RTs were recently inserted and could be still active in the *M. oryzae* genomes.

**FIG 1:**
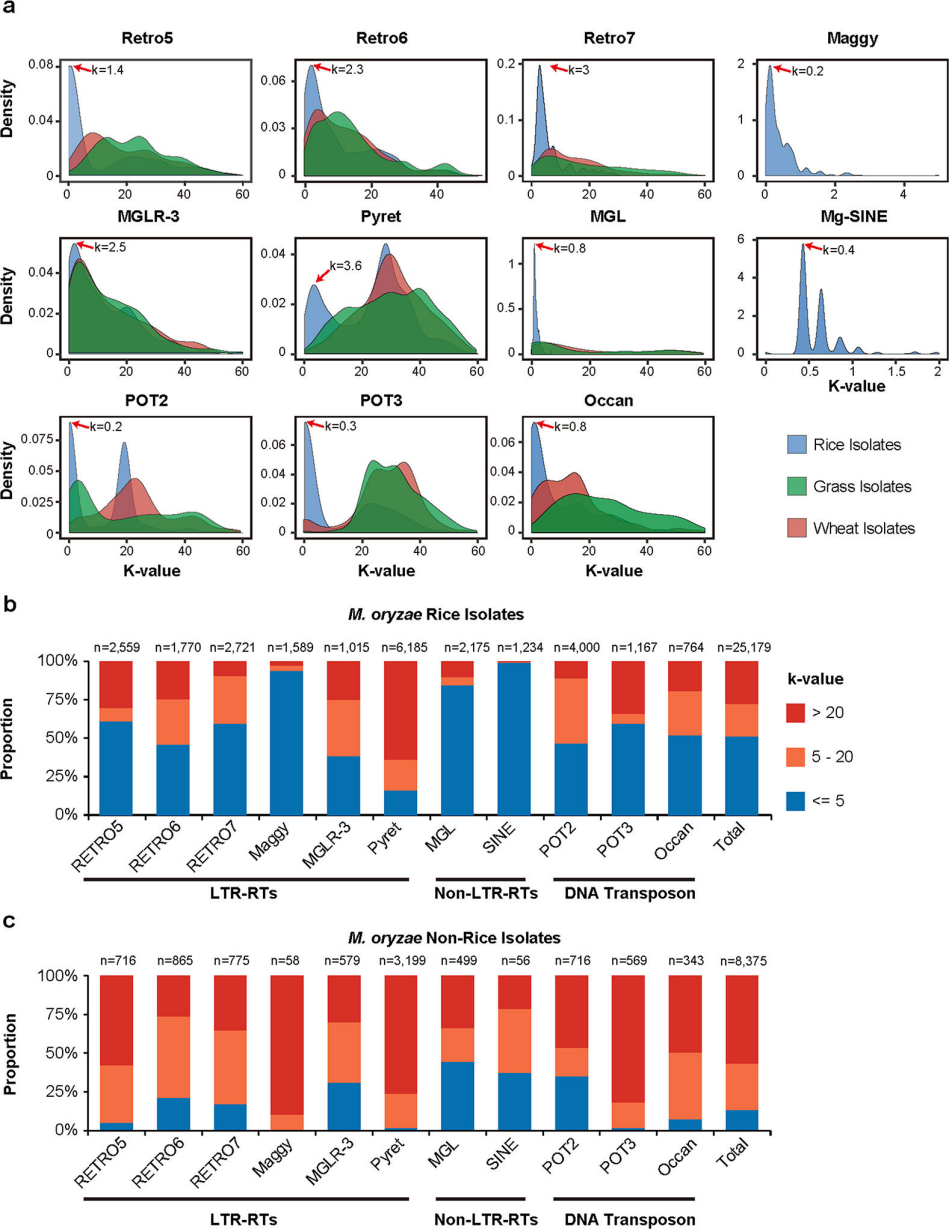
The distribution of 11 TE families in the *M. oryzae* population. (**a**) The Kimura 2-Parameter genetic distance of 11 TE families in three different *Magnaporthe* species were shown in density plot. (**b, c**) The *k*-value proportions of 11 TE families in *M. oryzae* rice isolates (**b**) and non-rice isolates (**c**). The numbers of TE fragment were marked at the top of bars.

Then, we analyzed 11 TE families that are most abundant in the genomes of *M. oryzae* rice isolates, including six LTR-RTs (RETRO5, RETRO6, RETRO7, Maggy, MGLR-3, and Pyret), two non-LTR-RTs (MGL and Mg-SINE), and three DNA transposons (POT2, POT3, and Occan) (Fig. S1) (12, 22–25). To assess the activity of these TE families, we calculated the Kimura 2-Parameter genetic distance (*k*-value) to measure the divergence between TE sequences and their associated consensus sequences (26). Low *k*-values indicate that the TE fragments were generated through recent insertion events, while high *k*-values indicate that the TE fragments are divergent copies generated through ancient transposition events (27). Our analysis revealed that all 11 TE families, especially sequences of MGL, Mg-SINE, Maggy, and a subset of POT2, POT3, and Occan, exhibit very low *k*-values, and more than half of all TE contents consisted of newly emerged TEs (*k*-values less than 5) (Fig. 1a and b), indicating a recent, large-scale burst of TEs in the genome of *M. oryzae* rice isolates. Notably, we observed two or more *k*-value peaks in POT2, Mg-SINE, and Pyret, indicating that these TEs families have undergone multiple rounds of amplification.

Furthermore, we investigated these TE families in 275 genomes of *M. oryzae* isolates (Table S1) (28, 29). We found that TEs only accounted for <5% of the genomes of non-rice isolates, which is dramatically lower than that found in *M. oryzae* rice isolates (Tables S1 and S2). Interestingly, the *k*-values of TEs were much larger and the proportion of newly emerged TEs was also much lower in *M. oryzae* non-rice isolates, indicating that the TEs were generated by more ancient insertion events and were inactive in *M. oryzae* non-rice isolates (Fig. 1b and c). Notably, the *M. oryzae* non-rice isolates contained only a few copies of fragmented Maggy and Mg-SINE, which were very abundant and possessed very low *k*-values in *M. oryzae* rice isolates, suggesting that the two TEs specifically amplified in *M. oryzae* rice isolates. In summary, our analysis demonstrated that the TEs were recently and specifically expanded in the genome of *M. oryzae* rice isolates and maintained high activity.

### Whole genome landscape of TE dynamics in *M. oryzae* population

**FIG 2:**
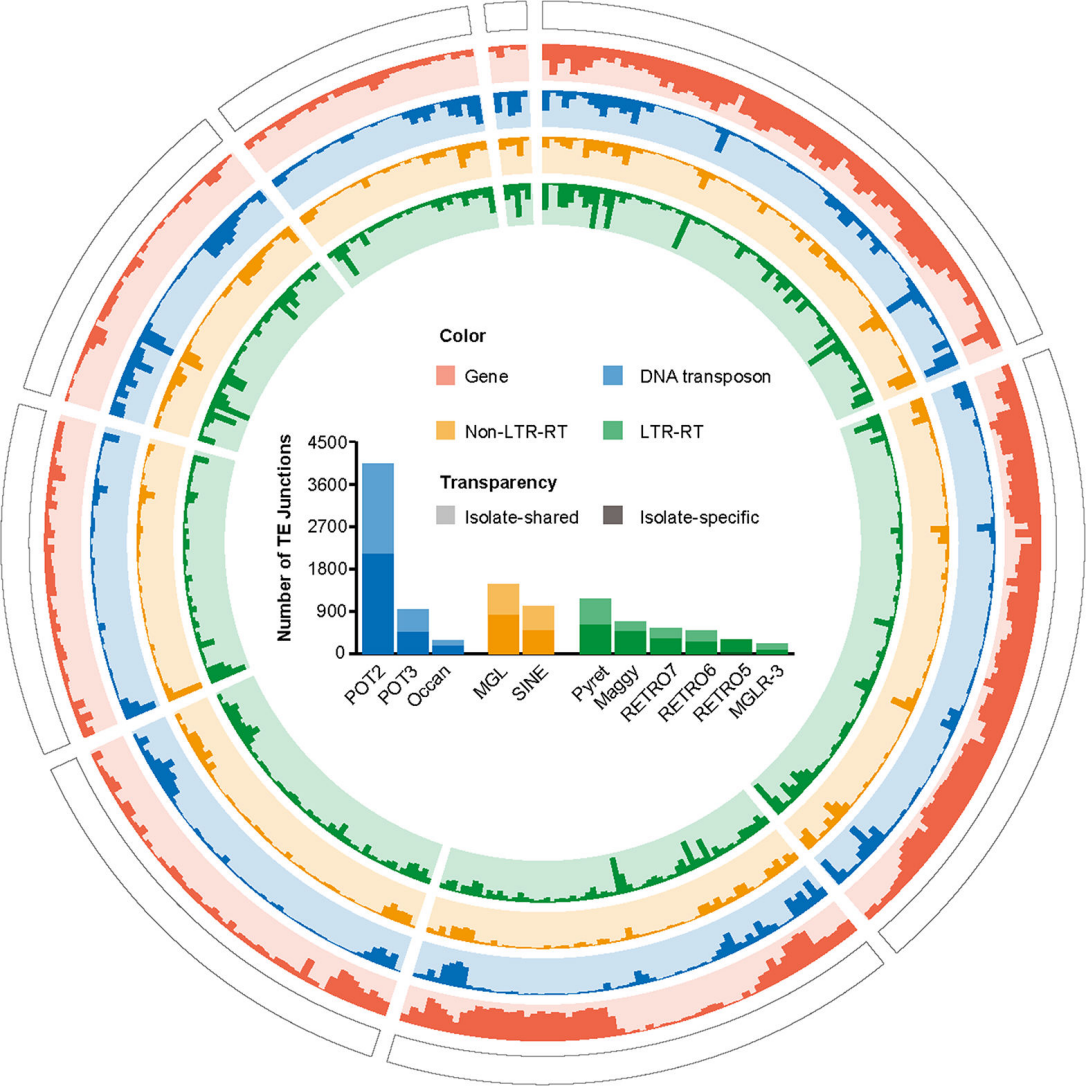
The number of TE junctions in the *M. oryzae* rice population. The circos diagram (from outer to inner) displays chromosomes, gene distribution, distribution of DNA transposon, Non-LTR-RT and LTR-RT junctions. The histogram shows the insertion site numbers of different TE families. The non-transparent and transparent colors represent the numbers of isolate-specific and isolate-shared TE junctions, respectively.

To examine the dynamics of TEs in the *M. oryzae* rice population, we conducted a genome-wide analysis of TE insertion sites in 90 rice isolates that had previously been published (30), with two *M. oryzae Setaria viridis* isolates as an outgroup (31). Using paired-end read mapping to the reference genome method, we identified a total of 11,163 TE insertion sites, with an average of 1,312 sites per isolate. To verify these insertion sites, 17 insertion sites randomly selected from Guy11 or FJ81278 isolates were proved to be presented as predicted through PCR-based (Polymerase Chain Reaction) genotyping or PacBio (Tables S3 and S4). The number (1,312 versus 739) and location of TE insertions differed dramatically between the rice and *S. viridis* isolates (Fig. S2), reflecting the evolutionary divergence of these subspecies and their corresponding TEs, which is consistent with the finding that these two subspecies diverged ~10,000 years ago (31). More than half (6,040/11,163) of the TE insertion sites were singletons specific to individual isolates (Fig. 2), indicating frequent TE transposition events. Notably, the number of POT2 insertion sites was substantially higher than those of other TE families, suggesting a higher activity and variability of POT2 in the *M. oryzae* rice population.

Furthermore, we conducted a comprehensive analysis of the genomic distribution of TE insertion sites in the *M. oryzae* rice population. Of the 11,163 TE insertion sites identified, 77% (8,582/11,163) was found to be located within 1 kb of the flanking regions of genes or intragenic regions, and over 40% of the genes (5,259/12,991) were embedded by these TE insertions. Our enrichment analysis showed that the distribution of the 11 TE families is non-random in the *M. oryzae* rice isolates, and each family displayed a distinct preference for specific genomic regions. For instance, Maggy, MGLR-3, RETRO5, RETRO7, Pyret, POT3, and Occan were predominantly distributed in intergenic regions, while POT2 displayed a preference for the gene flanking regions. Additionally, SINE, MGL, and RETRO6 were found to preferentially target intragenic regions (adjusted *P*-value < 0.01, Table S5). These findings provide valuable insights into the mechanisms underlying TE insertions and their potential impact on gene regulation in *M. oryzae*.

### Higher frequency of POT2 and POT3 insertions in promoter of SPs

**FIG 3:**
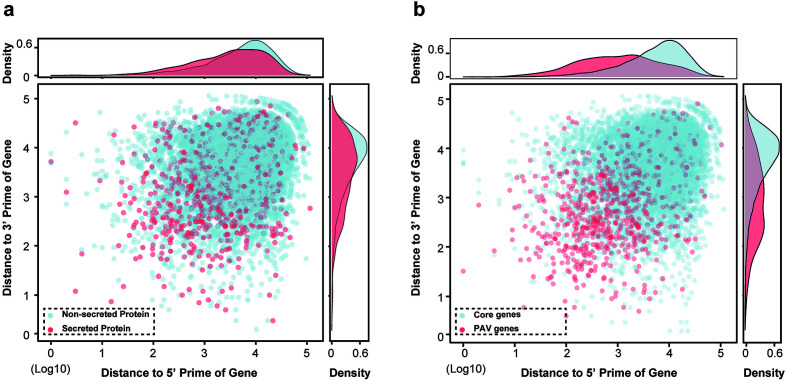
The TEs were in proximity to SPs and PAV genes. The distance of TEs to the 5′ and 3′ ends of SPs and non-SPs (**a**) or core genes and PAV genes (**b**).

We observed that genes encoding SPs were more closely associated with TE junctions (Fig. 3a), and the proportion of genes encoding SPs with TE insertion within 1 kb flanking regions was significantly (*P* = 7.6e−4) higher than in those of non-SPs (Table 1). Moreover, enrichment analysis for TEs associated with genes encoding SPs showed that POT2 and POT3 were overrepresented in promoters of genes encoding SP (adjusted *P*-value < 0.01, Table S6), implying that genes encoding SPs are more prone to disruption by POT2 and POT3.

**TABLE 1:** The numbers of SP and non-SP that have TE insertion in 1 kb flanking regions or within gene

	Total number	Embed	Upstream	Downstream	Total
Non-SP	11,445	2,247	1,978	1,893	4,414
SP	1,546	358	482	475	845
All	12,991	2,605	2,460	2,368	5,259

Previous studies have shown that genes with presence/absence variation (PAV) tended to be located near TEs in fungal pathogen genomes, while core genes were located further away from the TE-rich compartments (32–34). In this study, we compared the genomic distribution of core genes and PAV genes and found that PAV genes tended to be located closer to TE insertion sites than core genes (Fig. 3b). Our results were consistent with previous findings and suggested that PAV genes may be more susceptible to TE-mediated disruption, potentially contributing to their faster evolution in the context of host-pathogen interactions. Together, these findings suggested that TEs play a role in the evolution of pathogen effectors and contribute to the dynamic nature of host-pathogen interactions.

### Association of TEs with *M. oryzae* rice population divergence

**FIG 4:**
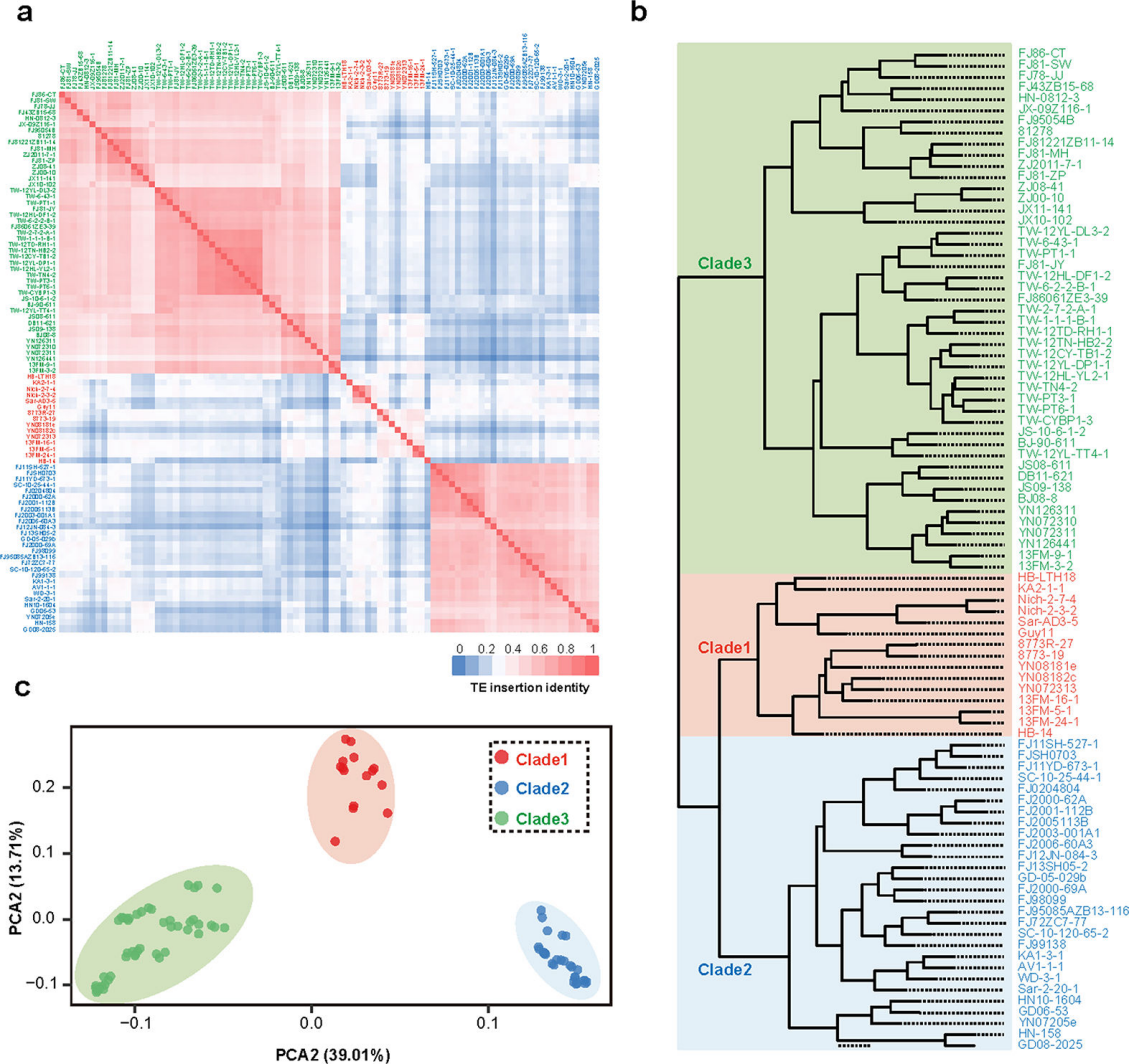
TE insertion was associated with the divergence of *M. oryzae* rice population. (**a**) The heatmap showing the identity of TE insertion site between each two isolates. (**b**) The hierarchical tree built by using the TE insertion sites of *M. oryzae* population. Three distinct clades were marked in red, blue, and green, respectively. (**c**) PCA using the TE insertion sites.

Considering that the *M. oryzae* rice population diverged within only 1000 years (30) and that the large-scale TE burst happened recently, we thus raised the question of whether the population divergence of *M. oryzae* is associated with recent TE amplification events. To characterize correlations between the 90 isolates, we estimated the distance for each of 2 isolates by calculating the identity of the TE insertion sites. The pairwise TE insertion identities varied from 17.6% between YN126441 and FJ12JN-084-3 to 87.2% between TW-PT3-1 and TW-PT6-1, with an average of only 38.7%, thereby strongly implicating the high variability of TE junctions between the different isolates. However, when we grouped these isolates based on the pairwise TE insertion identities, we discovered two distinct clusters (Fig. 4a) matching the Clade2 and Clade3 isolates that we had previously defined based on genome-wide SNPs (30). We noticed that the remaining isolates out of the two clusters were also able to match the Clade1 isolates even though they showed a relatively low pairwise TE insertion identity, which can be attributed to an earlier time of divergence of Clade1 isolates from the *M. oryzae* rice population when compared to the other two clade isolates. Furthermore, we found that the pairwise TE insertion identity between intra-clade isolates was higher than that between inter-clade isolates (Table S7). Surprisingly, the hierarchical tree constructed using TE insertion sites showed a high degree of similarity to the phylogenetic tree constructed based on whole-genome SNPs (Fig. 4b) (30), indicating that the recent TE amplification event has been a major force driving population divergence of *M. oryzae*. Consistently, principal component analysis (PCA) of these TE insertion sites displayed a similar pattern (Fig. 4c). Considering that the TEs were largely amplified recently in the *M. oryzae* genome (Fig. 1), we presumed that the recent high activity of TEs has been one of the major forces driving population divergence of *M. oryzae*.

### POT2 and Mg-SINE are critical for the divergence of Clade2 and Clade3 isolates

**FIG 5:**
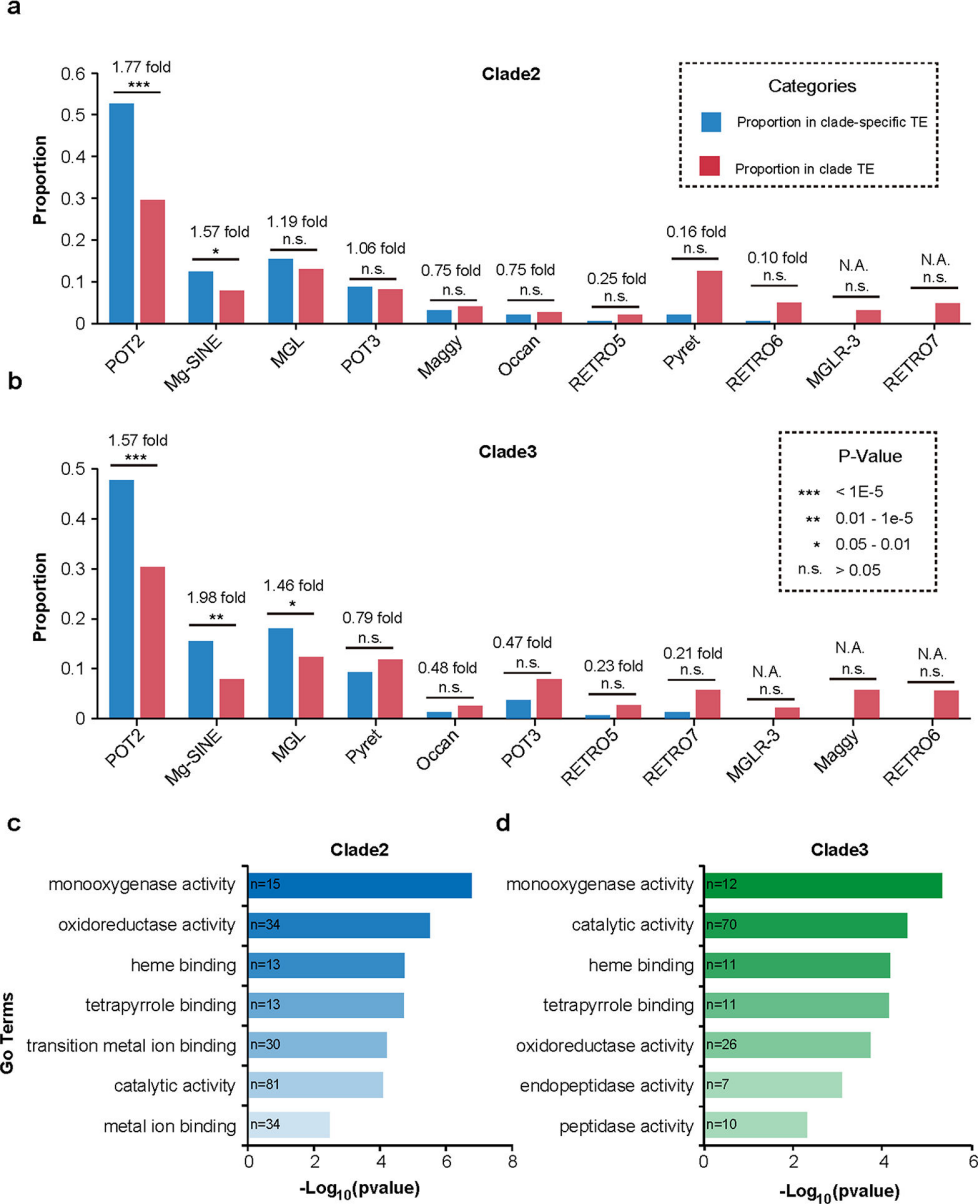
Characteristics of CSTs in the *M. oryzae* rice population. (**a, b**) Enrichment analysis of 11 TE families in Clade2- (**a**) and Clade3-specific (**b**) insertion sites. Fisher’s exact test was used for significance test. (**c**) GO enrichment analysis for the clade2- (**c**) and clade3-specific (**d**) TE-associated genes.

TEs are able to exert either beneficial or deleterious effects on host genomes, and the retention or elimination of TEs is largely determined by their impact on the host. Positive selection has been shown to drive the frequency of a TE locus to increase or decrease dramatically during a population bottleneck or in response to a new environment (35). We hypothesized that a portion of clade-specific TE (CST) insertion sites had been fixed in the intra-clade isolates, contributing to the adaptive evolution of the clade isolates. To identify the CST insertion sites, we empirically screened out those that were present in at least 80% of the intra-clade isolates and absent in more than 80% of the other two clade isolates. A total of 11 Clade1-specific, 212 Clade2-specific, and 168 Clade3-specific TE insertion sites were identified, with the number of Clade1-specific TE insertion sites being too small for subsequent analysis. Enrichment analysis revealed that POT2 and Mg-SINE TE families were overrepresented in both Clade2 and Clade3 data sets, indicating that the retention of insertion of these two TE families in subpopulations of *M. oryzae* rice isolates was tightly associated with the divergence of the rice-infection population (Fig. 5a and b).

We investigated the influence of CST junctions on genes, which, for this purpose, we referred to as CST-associated genes. We identified a total of 238 Clade2-specific and 173 Clade3-specific TE-associated genes. Interestingly, <10% of these genes overlapped, suggesting that the Clade2- and Clade3-specific TEs have distinct targeted genes (Fig. S2). Gene ontology (GO) enrichment analysis revealed that the Clade2- and Clade3-specific TE-associated genes were enriched under similar GO terms (adjusted *P*-value < 0.01, Fig. 5c and d). Of note, the top enriched term in both data sets was “GO:0004497, monooxygenase activity,” which is correlated to cytochrome P450s on the fungal genome. We further validated the GO enrichment results by performing enrichment analysis for these genes using the Pfam database (Table S8). Several previous studies have shown that fungal P450s possess detoxifying functions towards compounds produced by host plants during pathogen infection, thereby enhancing the fitness of the pathogenic fungus to specific host genotypes (31, 36–40). Therefore, we suggest that TE-induced variations in different members of P450s partially contribute to the differential pathogenicity of the two clade isolates.

### Genes associated with TE are significantly lower expressed

**FIG 6:**
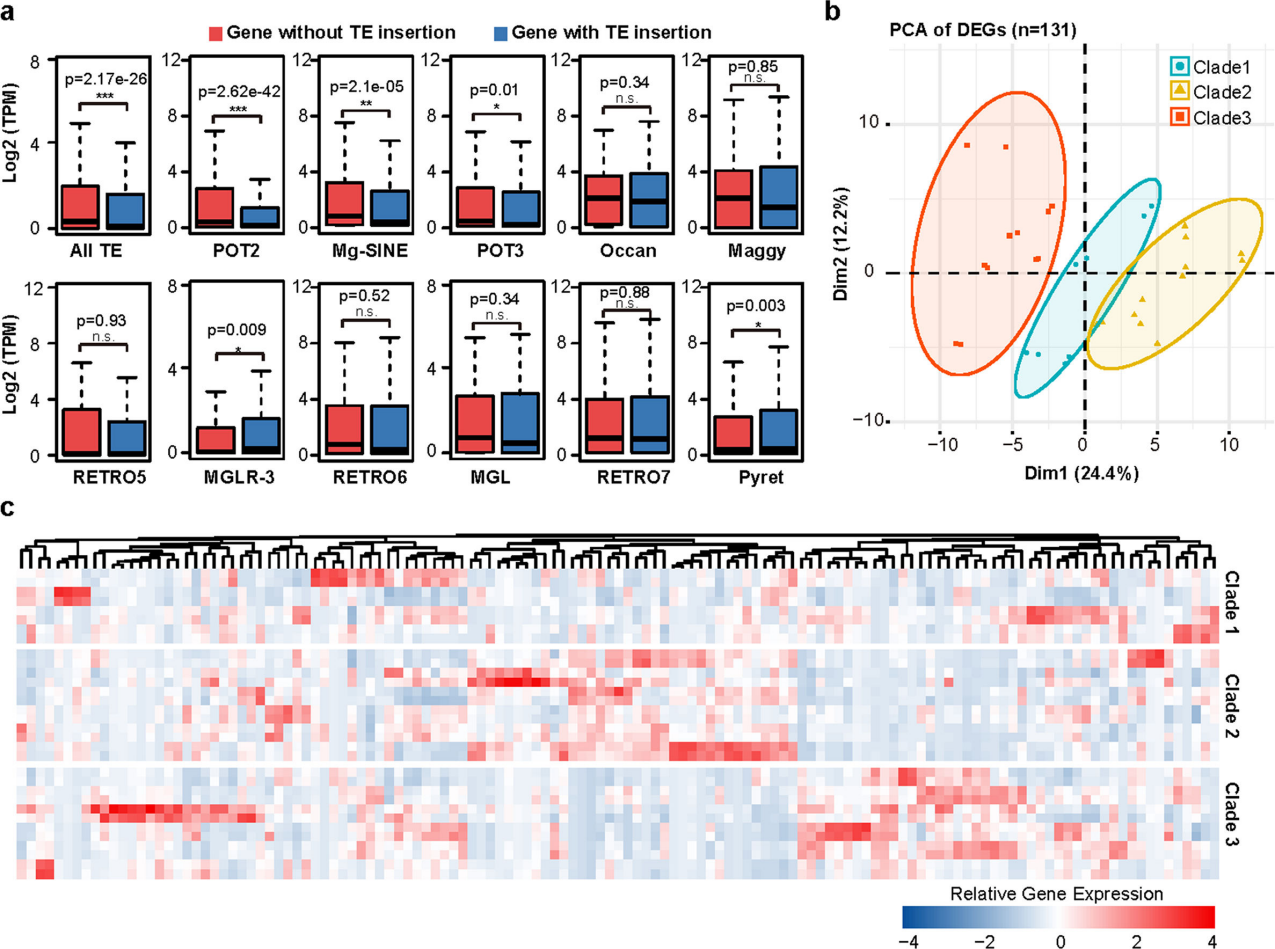
The impact of CST insertion on gene expression. (**a**) Comparison of gene expressions between the isolates present or absent with TE insertion. (**b**) PCA clustering of 16 isolates based on expression level of 131 clade-specific Differentially Expressed Genes (DEGs). (**c**) Heatmap showing expression level of 131 clade-specific DEGs in 16 isolates.

Previous studies have demonstrated that TEs were able to affect gene expression by inserting into gene promoters or intragenic regions (41–43). Therefore, we investigated whether TE insertion polymorphisms could shape the gene expression networks in *M. oryzae* rice populations. To this end, we selected 16 isolates and extracted total RNA for sequencing (Table S9). We identified 2,282 genes targeted by 4,236 TE insertion sites that were polymorphic between the 16 isolates. We then grouped the genes based on whether they contained TE insertion sites (TE-present or TE-absent) and compared the expression levels between these two groups. We observed that the TE-present gene group displayed significantly (*P* = 2.17e−26) lower expression levels than the TE-absent gene group (Fig. 6a), suggesting that TEs have negative effects on the expression of their target genes. We have identified 131 genes that exhibited clade-specific expression patterns. Furthermore, considering the expression levels of these genes, we were able to divide 16 isolates into 3 distinct clusters using principal component analysis (PCA). These findings suggest that TE-mediated gene regulation had a profound impact on population divergence (Fig. 6b and c). Interestingly, we found that only genes with insertion polymorphisms of POT2 and Mg-SINE displayed transcriptional suppression, while other TE families displayed little impact on the expression of their target genes. Our results suggest that the insertion polymorphisms of POT2 and Mg-SINE were able to shape the gene expression network of *M. oryzae* by inducing transcriptional suppression.

### *CST6* and *CST10* were required for the pathogenicity of *M. oryzae*

**FIG 7:**
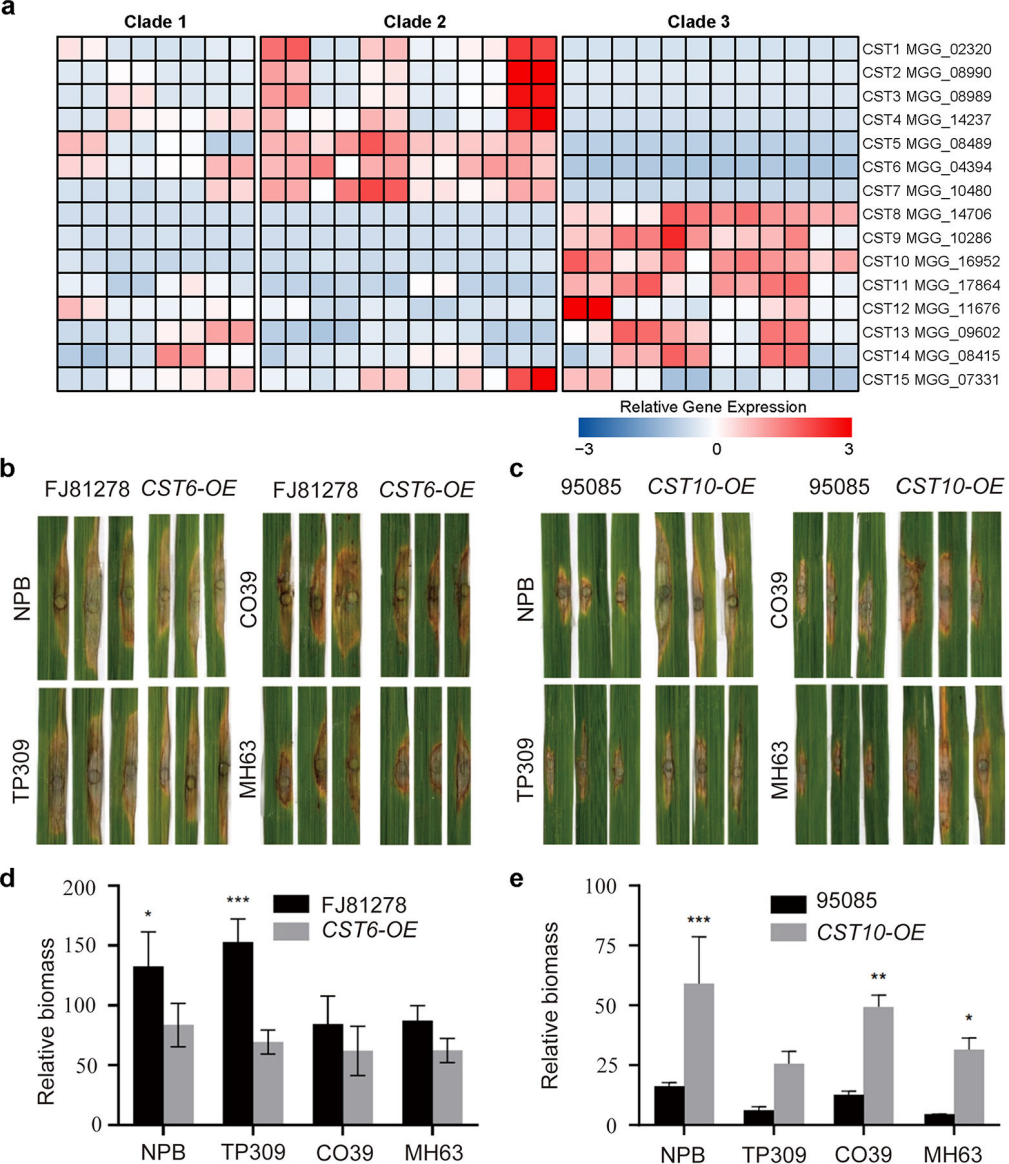
CST6 and CST10 were required for the pathogenicity of *M. oryzae* rice isolates. (**a**) Heatmap showing expression level of 15 CST-associated genes (CSTs) in 16 isolates. (**b**) Leaf punch inoculation assays were conducted to assess the impact of CST6 overexpression on two Japonica cultivars (NPB and TP309) and the two Indica cultivar (CO39 and MH63). The Clade3 isolate (FJ81278), lacking CST6 expression, was used as the wild-type control. (**c**) Leaf punch inoculation assays were conducted to assess the impact of CST10 overexpression on two Japonica cultivars (NPB and TP309) and the two Indica cultivar (CO39 and MH63). The Clade3 isolate (95085), lacking CST10 expression, was used as the wild-type control. (**d**) Relative fungal biomass on leaf punch inoculation assays of CST6 overexpression on two Japonica cultivars (NPB and TP309) and the two Indica cultivar (CO39 and MH63). (**e**) Relative fungal biomass on leaf punch inoculation assays of CST10 overexpression on two Japonica cultivars (NPB and TP309) and the two Indica cultivar (CO39 and MH63).

To further investigate the impact of CST in the virulence divergence within *M. oryzae* rice population, we selected 15 of the CST-associated genes (CSTs) for functional analyses (Fig. 7a; Table S10). We defined CSTs as genes that have TE insertion exclusively present in one clade and absent in another clade. Among them, CST1-7 are Clade3-specific, and are only transcriptionally active in Clade2 isolates, while CST8-15 are Clade2-specific, and are only transcriptionally active in Clade3 isolates. We amplified CST1-7 from a Clade2 isolate, 95085, and ectopically overexpressed them in a Clade3 isolate FJ81278. Conversely, CST8-15 were amplified from a Clade3 isolate, FJ81278, and ectopically overexpressed in a Clade2 isolate 95085 (CST8-11) or transiently expressed in tobacco leaves (CST12-15). The functional analyses revealed a role of CST6 and CST10 in the pathogenicity of *M. oryzae* by leaf punch inoculation assays on the two Japonica cultivars (NPB and TP309) and the two Indica cultivar (CO39 and MH63) (Fig. 7b and c). While the CST6-OE strain produced smaller lesions and reduced fungal biomass compared with the wild type, suggesting a potential role as a negative regulator of virulence (Fig. 7b and c). The CST10-OE strain was more aggressive and produced larger lesions and increased fungal biomass compared with the wild type, suggesting its function as a positive regulator of virulence (Fig. 7d and e). These results suggest that CST can profoundly impact virulence in *M. oryzae*, influencing its interaction with different rice species.

## DISCUSSION

The high genomic plasticity and rapid evolution of the plant pathogen *M. oryzae* present a severe challenge for rice blast disease control (44, 45). Previous studies have identified transposons as a major driving force for the adaptive evolution of fungal pathogen genomes (10). Insertion polymorphisms of TEs have been shown to lead to genomic instability, increased chromosomal recombination, and accelerated gene evolution (17, 46). However, the precise role of TE dynamics during *M. oryzae* evolution has remained poorly understood. To address this, we conducted a population-scaled TE analysis of *M. oryzae* to investigate how TE insertion polymorphisms contributed in shaping population structure and divergence.

Previous study estimated that the *M. oryzae* rice population diverged 175–2,700 years ago (45). Here, we employed two methods to assess the activity of TEs in the genome of *M. oryzae*. First, we estimated the insertion time of LTR-RTs and secondly, we calculated the Kimura 2-parameter genetic distance for the 11 most abundant TE families. Both analyses indicate that TEs have undergone recent amplification in the *M. oryzae* genome and have remained highly active. Previous studies have suggested that the insertion of TEs having caused gene PAVs to be the main evolutionary mechanism driving the divergence of host-specific *M. oryzae* lineages (16). In this study, we investigated the abundance and activity of 11 TEs in pathogens from wheat and grass infecting lineages, and found that these 11 TEs have undergone specific expansion in *M. oryzae* rice isolates while remain inactive in non-rice isolates. On the account of results, we propose that the recent burst of TE activity could be the primary factor responsible for the divergence of *M. oryzae* from other host infecting lineages and rice subspecies.

To elucidate the role of TEs in genome evolution and divergence, we conducted a genome-wide survey of TE insertion sites in 90 *M*. *oryzae* rice isolates (30). We utilized PoPoolationTE2, which is a validated method for estimating TE insertion frequency in populations (47). The TE insertion junctions varied widely among the 90 isolates, indicating the highly dynamic and active feature of TEs in *M. oryzae* rice populations. Consistent with prior research, we found that the distribution of TE junctions across the genome was not evenly distributed, and different TE families or superfamilies displayed preferences for insertion into distinct genomic regions (7, 17, 19).

TEs have been shown to be a major contributor in causing genomic variations, and the evolution of plant pathogens (19, 48, 49). Previous studies have reported that in the major wheat pathogen *Zymoseptoria tritici*, recent TE bursts were associated with the proximity to genes (48). And TE insertion repressed the expression of *REP9-1* (49) or *Avr3D1* (50), and consequently resulted in the altered virulence in different isolates of this fungus. In the polyphagous fungal pathogen *Rhizoctonia solani*, TEs mediated the structural variations of regions encoding pathogenicity associated genes (51). Similarly, TE insertions in or around *Avr* genes in *M. oryzae* were able to lead to transcriptional silencing and loss of avirulence function (14, 52–56). Our analysis revealed that TE junctions are frequently observed in the flanking regions of genes encoding secreted proteins (SPs), and the proportion of genes encoding SPs with TE insertions is higher than that of non-SPs. This is consistent with previous findings that SPs are enriched in repeat-rich regions and are prone to rapid evolution (13, 57). We propose that the variation in SPs, induced by TE insertion polymorphisms, is able to facilitate adaptive evolution of *M. oryzae*. PAVs in genes resulting from TE insertions have been identified to be associated with the divergence of host-specific *M. oryzae* lineages (16). Consistent with this, we found that genes exhibiting high gain/loss polymorphisms in the 90 isolates were preferentially located near TE junctions, suggesting that TE-mediated PAV constitutes a significant mechanism underlying differentiation of host-specific or intra-species *M. oryzae* rice isolates.

Through the comparison of TE junctions in 90 *M*. *oryzae* rice isolates, we observed a clustering pattern that was similar to the three previously defined *M. oryzae* clades. This led us to investigate the potential role of TEs in *M. oryzae* rice population divergence. We constructed a hierarchical tree based on the TE junctions and found that it closely resembled the phylogenetic tree constructed using genome-wide SNPs. PCA of the TE junctions also yielded a similar clustering pattern. These findings suggested that the transposition of TEs was strongly associated with *M. oryzae* rice population divergence. Given that both the junction of the majority of TEs in the *M. oryzae* rice isolate genomes and the divergence of *M. oryzae* rice population were occurred recently, we hypothesize the recent burst of TEs to be a driving force contributing to the *M. oryzae* rice population divergence.

TE loci that undergo positive selection during evolution will be retained and will exhibit high frequencies in a population. Taking into account the hierarchical tree and PCA results, we postulate that the intra-clade isolates have a fixed set of TE insertion sites that are specific to each clade. These CST insertion sites are then able to serve as molecular markers to distinguish isolates belonging to the three clades. Interestingly, we found that POT2 and Mg-SINE were enriched in CST insertion sites, suggesting that these clade-specific insertions of POT2 and Mg-SINE were beneficial for the adaptive evolution of clade isolates. Furthermore, we noted that cytochrome P450 proteins were overrepresented in both Clade2- and Clade3-specific TE-targeted genes. Given the essential roles of P450s in detoxifying phytoalexins produced by host plants, we hypothesize that the differential pathogenicity of clade isolates may be partially due to the variations in P450s induced by CST insertions.

TEs integrated within or flanking genes have been shown to induce gene silencing through the formation of heterochromatic regions (10, 43). To investigate the correlation between TE insertion and gene expression regulation in *M. oryzae*, we performed RNA sequencing on 16 isolates from the 3 clades and conducted functional studies on genes disrupted by CSTs. Our analysis revealed that genes containing a TE insertion displayed significantly lower expression compared to genes without TE insertion. Notably, only POT2 and Mg-SINE insertions led to substantial suppression of gene expression. The initial functional analysis suggests that these CSTs play a crucial role in the pathogenic process. Surprisingly, these CSTs exhibit dual functionality, acting as both negative and positive regulators of virulence, while the detailed mechanisms underlying these roles require further investigation. Therefore, we hypothesized that clade-specific insertions of POT2 and Mg-SINE contribute to the adaptive evolution of clade isolates by regulating expression of specific genes and affecting adaptive traits.

Through a comprehensive analysis of TEs in populations of the rice-infecting fungus *M. oryzae*, we have revealed the crucial roles played by recent TE insertional bursts in the adaptive evolution and diversification of this fungal lineage. We demonstrate that recent TE insertions have led to the emergence of genes with clade-specific expression patterns, contributing significantly to the divergence of *M. oryzae* rice population and their adaptation to different rice subspecies. These findings highlight the significance of TE-mediated genetic changes in the regulation of gene expression, which in turn contributes to clade divergence and allows *M. oryzae* to adapt to diverse environmental pressures, including those imposed by different rice cultivars. Our findings highlight the significance of TE-mediated genetic changes in the regulation of gene expression, which in turn contributes to clade divergence.

## MATERIALS AND METHODS

### RNA extraction, library generation, and sequencing

The fungal strains were cultured in liquid complete medium by incubation at 28°C under shaking at 110 rpm for three days. The mycelium was filtered, washed with double-deionized water, and dried before being ground in liquid nitrogen. Ground samples were transferred into DNase/RNase-free Eppendorf tubes, suspended in 1 mL Trizol, and vortexed vigorously. To eliminate proteins, 200 µL of chloroform was added to the mixture, which was then shaken for 15 s. After centrifugation at 12,000 rpm for 15 min at 4°C, 400 µL of the supernatant was collected and mixed with 400 µL of cold isopropanol. The mixture was kept at −20°C for at least 2 h, then centrifuged at 12,000 rpm for 15 min at 4°C. The supernatant was discarded, and the precipitates were washed with 1 mL of 70% alcohol and centrifuged at 12,000 rpm for 5 min at 4°C. After air drying for 5 min at room temperature, the pellets were diluted with 54 µL of DNase/RNase-free deionized water and treated with 2 µL of DNase I at 37°C for 30 min. The mixture was brought up to 800 µL with RNase-free water, followed by the addition of an equal volume of chloroform. After gentle mixing, the mixture was centrifuged at 12,000 rpm for 15 min at 4°C. About 500 µL of the supernatant was collected and mixed with 500 µL of cold isopropanol. The mixture was then kept at −20°C for more than 3 h, followed by centrifugation at 12,000 rpm for 15 min at 4°C. The precipitates were washed with 1 mL of 70% alcohol, air-dried for 5 min at room temperature, and eluted with DNase/RNase-free deionized water. The RNA samples were then stored at −80°C for RNA sequencing analysis.

### Estimation of TE activity

LTR-Finder (58) with modified parameters of “-D 15000 -d 1000 L 7000 L 100 p 20 C -M 0.8,” LTR-harvest (59) with modified parameters of “-similar 80 -vic 10 -seed 20 -seqids yes -minlenltr 100 -maxlenltr 7000 -mintsd 4 -maxtsd 6 -motif TGCA -motifmis 1” and LTR-Retriever, and LTR-Retriever (60) with default parameter was used for *de novo* identification of full-length LTR-RTs and estimation of insertion time. Information on TE classification is based on previous research (12) and the conserved domains of TE consensus sequences were predicted by Conserved Domain Database (61). The Kimura 2-Parameter genetic distances (*k*-values) of TE fragments were calculated by RepeatMasker (62) with option “-a”.

### Prediction of secreted proteins and PAV genes

To identify putative secreted proteins, several criteria were employed, including the presence of a signal peptide cleavage site, the absence of a transmembrane domain, and a protein length of <400 amino acids. SignalP 4.0 and TMHMM 2.0 were utilized for signal peptide and transmembrane domain prediction, respectively (63, 64). The transcript sequences of the 70–15 reference genome were aligned to the previously published assemblies of 90 isolates using NCBI-blastn (National Center For Biotechnology Information) with default parameters. Genes that exhibited more than 90% similarity with no gaps longer than 50 bp when compared to the assemblies were marked as “present”. Genes that did not meet these criteria were marked as “absent”. PAV (presence-absence variation) genes were defined as those that were absent in more than five isolates, while core genes were defined as those present in all isolates.

### Identification of TE insertion sites in *M. oryzae* populations

*M. oryzae* 70–15 TE is annotated by RepeatMasker with 11 TEs as TE library. The pair-end reads were mapped to the *M. oryzae* 70–15 reference genome using BWA (Burrow-Wheeler Aligner) (65) with default parameters. The alignment files were exported to PoPoolation2 (66) with default parameter to identify TE insertion site for each isolate. The TE insertion sites with score less than 0.3 were filtered, and the insertion sites located within 50 bp were considered as one insertion event.

### Construction of TE hierarchical tree, PCA

The insertion sites that are present in at least five isolates were used for constructing hierarchical tree and PCA. The TE insertion sites in PAV format were exported to the R package “hclust” (67) for construction of TE hierarchical tree. Vcftools (68) and Plink (69) were used for PCA.

### Functional enrichment analysis

GO annotation information of *M. oryzae* was obtained from the JGI database (70). Conserved domains of *M. oryzae* protein sequences were predicted using the Pfam database (71). Fisher right-tailed test was used for enrichment analysis and a cutoff *P*-value < 0.05 was used to define significant enrichment.

### RNA-seq analysis

The clean reads were mapped to the 70–15 reference genome using hisat2 v2.2.1 (72) with default parameters. Stingtie v2.1.4 was used for expression quantification (73). Gene expressions were normalized with transcripts per million (TPM). The expression of each gene in the isolates with or without TE insertion was counted, averaged, and compared. Two-tailed Wilcoxon paired test was used to estimate the significance of expression difference.

### Pathogenicity assay of the *CST*-overexpressing transformants

The respective coding sequence (CDS) of *CST6* and *CST10* were amplified from the Clade2 isolate, 95085, and the Clade1 isolate, FJ81278, respectively, and were inserted into the *pKNT-RP27* vector. The resulting constructs were then introduced into FJ81278 (*CST6*) or 95085 (*CST10*), respectively. The polyethylene glycol-mediated protoplast transformation was performed as described previously (74). To determine the role of selected *CST* genes in the pathogenicity of *M. oryzae*, punch inoculation was performed as previously described (75). In brief, 10 µL spore solution (5 × 10^5^ spores/mL in sterilized water containing 0.02% Tween) was inoculated into wounded rice leaves. The inoculated rice plants were placed in a greenhouse. Disease symptoms were recorded 10 days post inoculation. DNA extracted from the diseased rice leaves was subjected to the quantitative fluorescence analyses. The primers used in this study were listed in Table S11 .

## Data Availability

All high-throughput sequencing data generated in this study are accessible at NCBI’s Gene Expression Omnibus (GEO) via GEO Series accession number GSE205351. The consensus TE sequnces of M. oryzae (https://github.com/S-t-ing/mBio-data-availablility/blob/main/Mo.TE_Consensus.fasta), Genome-wide annotation of TEs in the genomes of 275 Magnaporthe isolates (https://github.com/S-t-ing/mBio-data-availablility/tree/main/gff), and the TE insertion sites among the 90 Magnaporthe rice isolates (https://github.com/S-t-ing/mBio-data-availablility/blob/main/TE%20insertion%2090%20isolates.xlsx) were available online.

## AFTER EPUB

*[This article was published on 27 March 2024 with typographical errors in Results. The text was corrected in the current version, posted on 10 April 2024.]*

## SUPPLEMENTAL MATERIAL

Supplemental figures mbio.00086-24-s0001.docx Fig. S1-S3.

Table S1 mbio.00086-24-s0002.xlsx The proportions of 11 TE families on the genomes of 275 *M. oryzae* isolates.

Supplemental tables mbio.00086-24-s0003.docx Tables S2-S9.

TABLE S6 mbio.00086-24-s0004.xlsx Basic information about 15 clade-specific TE-associated genes (CSTs).

TABLE S7 mbio.00086-24-s0005.xlsx Primers used in this study.

ASM does not own the copyrights to Supplemental Material that may be linked to, or accessed through, an article. The authors have granted ASM a non-exclusive, world-wide license to publish the Supplemental Material files. Please contact the corresponding author directly for reuse.
