# Supplementary material for: Transposable elements impact the population divergence of rice blast fungus Magnaporthe oryzae
Source: mBio. 2024 Mar 27;15(5):e00086-24. doi: 10.1128/mbio.00086-24 (PMC11077969; doi:10.1128/mbio.00086-24)
Supplement: Supplemental figures — Fig. S1-S3. [file mbio.00086-24-s0001.docx]

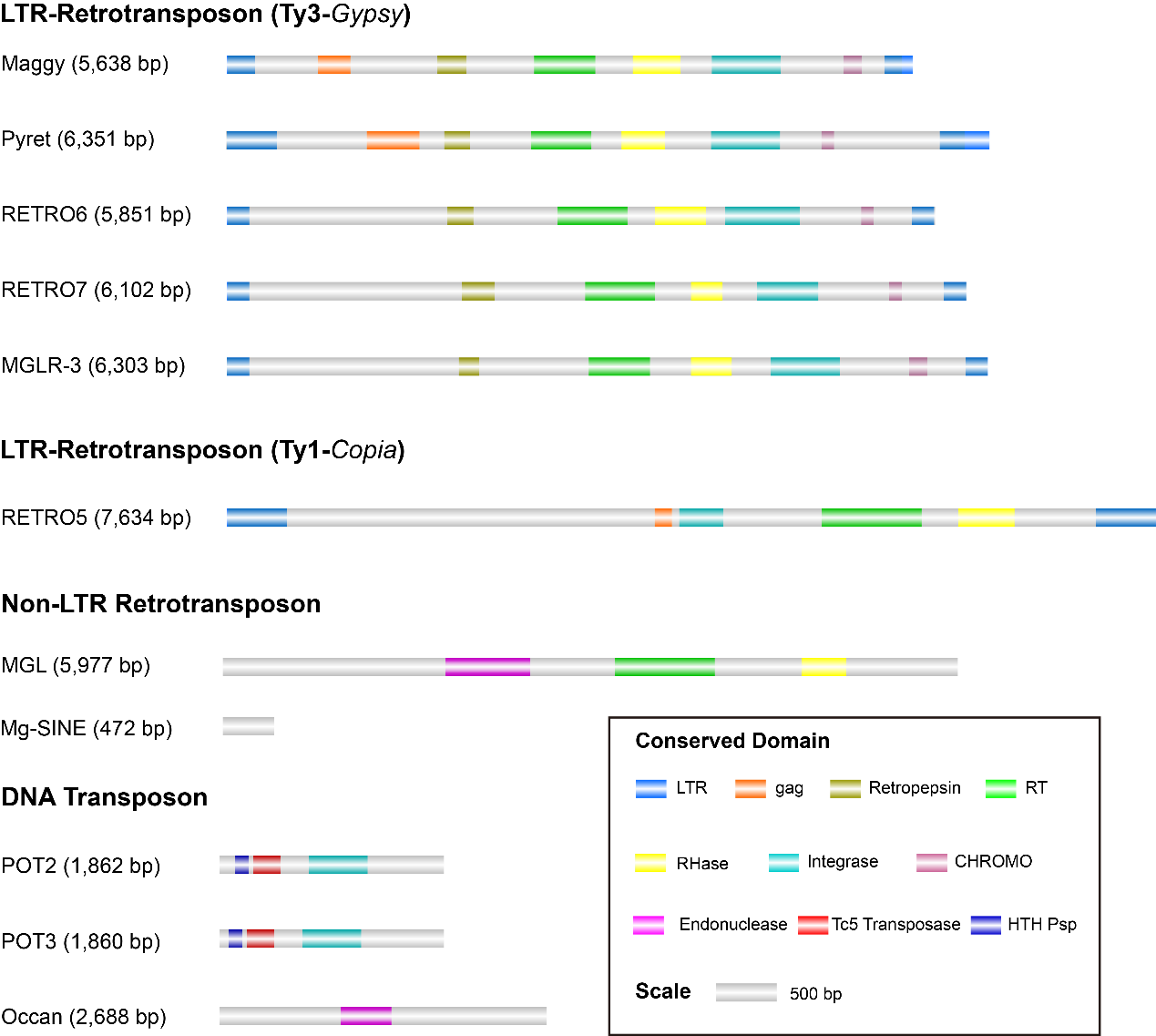


**FIG S1** Schematic of the 11 most abundant TE families on the genome of the *M. oryzae* rice isolate. The conserved domains in the relative location of the TE consensus were highlighted using different colors.


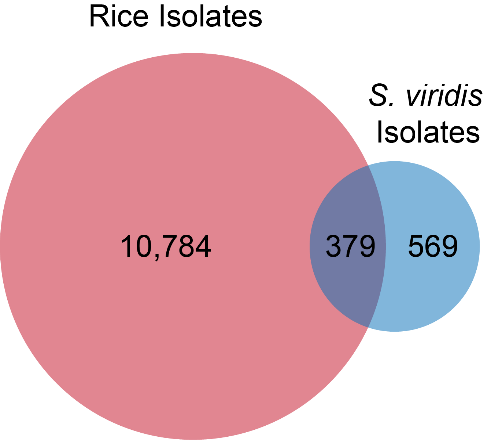


**FIG S2** The venn chart compares the TE insertion loci of 90 *M. oryzae* rice isolates and two *S. viridis* isolates.


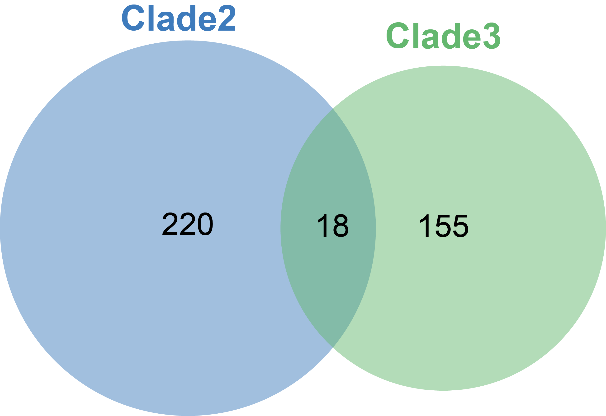


**FIG S3** The venn chart displays the intersection of clade2-specific and clade3-specific TE-associated genes.
